# The state of clinical education and factors affecting effective clinical education: the point of view of nursing and midwifery students

**DOI:** 10.1186/s12909-023-04957-z

**Published:** 2023-12-15

**Authors:** Mohammadreza Asadi, Sajad Noorian, Sanaz Motefakker, Fatemeh Heydari, Neda Shahsavari, Mojtaba Senmar

**Affiliations:** 1 Social Determinants of Health Research Center, Research Institute for Prevention of Non–Communicable Diseases, Qazvin University of Medical Sciences, Qazvin, Iran; 2 Department of Statistics, Faculty of Science, University of Qom, Qom, Iran

**Keywords:** Clinical, Education, Midwifery, Nursing, Student

## Abstract

**Background:**

Clinical education is the basis of education in medical sciences. Clinical education, as the essence of education in nursing and midwifery, promotes social health, improves health care, and reduces mortality. Considering the position of nursing and midwifery, investigating the views of students in this field can be an effective step in improving clinical education. Therefore, the present study was conducted to investigate the status of clinical education and the factors affecting effective clinical education from the point of view of nursing and midwifery students.

**Methods:**

A descriptive-analytical study was conducted among nursing and midwifery students at Qazvin University of Medical Sciences in 2022–2023. Using available sampling, 242 students were included in the study. Students were included in the study if they completed at least one unit of in-person internship. Refusing to continue the study for any reason and having a practical nurse certificate were the criteria for exclusion from the study. The data collection tools included a demographic information questionnaire, a questionnaire to assess the status of clinical education, and a questionnaire on factors affecting effective clinical education. The data were analysed with descriptive and inferential statistics and SPSS 20 software.

**Results:**

The mean age of the participants in this study was 21.66 ± 2.25. A total of 180 (74.4%) of the participants were women, and the rest were men. The results showed that the general condition of clinical education is at an average level (103.16 ± 19.21). It was also found that the clinical education status of midwifery students was better than that of nursing students, and this difference was significant (*p* = 0.003). Among the fields of clinical education, the highest score belonging to the field of objectives and planning was reported on the average level (34.39 ± 6.66). Among the factors affecting effective clinical education, the highest score was given to the field of personal characteristics of the student (33.97 ± 5.99). The results showed that there is a significant relationship between the grades of the general state of clinical education with the academic semester (*p* = 0.001) and interest in the field of study (*p* < 0.040).

**Conclusions:**

Based on the findings of the present study, clinical education is at an average level. Among the factors affecting effective clinical training, the field of personal characteristics of the student is more effective in clinical training. Providing educational facilities according to the number of students, using modern teaching methods, and determining and communicating the duties of professors and students can help to improve clinical education.

## Background

Nursing and midwifery are two professions consisting of science and art in medical sciences [1, 2]. As important healthcare groups, they make up 59% of the total employees of the health system [3, 4]. With the development of societies and the need for a nursing and midwifery workforce, education is essential to prepare nursing and midwifery students to enter healthcare environments [5, 6]. Clinical education is a key part of education in nursing and midwifery [7]. These students spend more time in the clinical environment than in the classroom during their education [8]. Clinical education, as the essence of education in these two disciplines, has led to the promotion of social health, improvement of health care, and reduction of maternal and infant mortality and causes students to apply theoretical training in the direction of patient care at the bedside [7, 9, 10]. However, clinical education provides an opportunity for students to transform theoretical knowledge into various mental, psychological, and motor skills that are necessary for patient care [11].

Clinical education is the foundation of education in medical sciences [12], and it is noteworthy that increasing the quality of clinical experiences and skills in students requires effective clinical training [13]. Although the learning of students in the clinical environment depend on various factors [14], effective clinical education is a prerequisite for the correct provision of care measures by students [12]. Therefore, clinical training can improve critical thinking and decision-making skills and increase self-confidence among students [15]. Since guiding students to achieve clinical goals requires identifying effective behaviors and factors in clinical education, effective clinical education has been the focus of many researchers in recent years [12]. While the gap between theoretical and practical training does not give the student the necessary opportunity to acquire clinical skills [16], it must be said that students as recipients of educational services are the best source for identifying clinical education problems [17].

In general, the importance of clinical education is considered in all texts as a vital and necessary element for the continuous growth of students [18]. In such a way, its enhancement will improve the quality of nursing and midwifery care [13]. Eliminating existing deficiencies and improving the status of clinical education depends on determining the status of clinical education and identifying the factors affecting effective clinical education. Due to the different professors, learning environments, educational systems, and facilities in each university unit, the present study was conducted to investigate the clinical education situation and identify the factors affecting effective clinical education from the point of view of nursing and midwifery students.

## Methods

### Setting and study design

This cross-sectional study was conducted in 2022–2023 among midwifery and nursing students at Qazvin University of Medical Sciences, Iran. By choosing α = 0.05 (confidence coefficient 0.95) and Β = 0.20 (test power 80%) and choosing *r* = 0.2 using the following formula.

$$n=\frac{\left(z_{1-\alpha/2}+z_{1-\beta}\right)^2}{r^2}+3$$

The minimum sample size was 199 people. Finally, 242 people were included in the study. Sampling was performed by available methods. Students were included in the study if they completed at least one unit of in-person internship. Refusing to continue the study for any reason and having a practical nursing certificate were the criteria for exclusion from the study. Questionnaires were provided to students on different days of the week. If needed, more information was given to the students. All questionnaires were completed in the presence of the researcher. Participants were allowed to refuse to answer some items or withdraw from the study without consequences.

### Data collection tools

Part 1: To collect demographic information, a researcher-made checklist was used. This checklist included age, sex, marital status, place of residence, year of entry into university, interest in the field, academic semester, grade point average, and other educational qualifications.

Part 2: Questionnaire for examining the clinical education status.

The clinical education status survey questionnaire included 33 questions in the form of a 5-point Likert scales as excellent, good, average, poor, and very poor, with the highest score being "5" and the lowest score being "1" in 5 fields. The first field, goals and educational program include 11 questions with a score range of 11 to 55.41 to 55 good levels, 26 to 40 average levels, and 11 to 25 poor levels. The second field of the instructor's performance includes 9 questions with a score range of 9 to 45. A score of 35 to 45 is a good level, a score of 22 to 34 is an average level, and a score of 9 to 21 indicates a poor level. The third field of interacting with students includes 4 questions with a score range of 4 to 20: 16 to 20 at a good level, 10 to 15 at an average level, and 4 to 9 at a poor level. The fourth field of the educational environment includes 5 questions with a score range of 5 to 25.19 to 25 good levels, 12 to 18 average levels, and 5 to 11 poor levels, and the last field, the monitoring and evaluation field, includes 4 questions with a score range and points similar to the third field. The validity and reliability of the above tool have been confirmed in a study at Ahvaz University of Medical Sciences. The reliability of the questionnaire was investigated by the test–retest method, and the correlation coefficient between the two times was reported to be 0.88 [12, 16]. The internal reliability of the questionnaire in the present study was 0.87 with Cronbach's alpha.

#### Section 3. Questionnaire of factors affecting effective clinical education

The questionnaire for examining the factors affecting effective clinical education included five general fields: personal characteristics of the student (9 questions), personal characteristics of the clinical Instructor (6 questions), clinical environment conditions (5 questions), educational planning (6 questions) and clinical evaluation (6 questions). The 32 questions of this section were rated based on a Likert scale from very little to very much and scored from 1 to 5. In the whole questionnaire, the maximum score that can be obtained is 160, and the minimum score is 32. The validity and reliability of this questionnaire were checked and confirmed by Khemtizare et al. in Ahvaz University of Medical Sciences with a sample size of 118 students and 28 faculty members. The reliability coefficient of the questionnaire has been reported as 0.94 by calculating the internal consistency index (Cronbach's alpha) [12, 19]. In the current study, the internal reliability of the questionnaire was good (Cronbach's alpha = 0.89).

### Analysis

Data analysis was performed by SPSS version 26 software using descriptive (mean and standard deviation) and inferential statistics. Independent two-sample t tests and Kruskal Varis tests were used to compare the mean scores in the nursing and midwifery groups. Pearson's correlation coefficient was used to check the relationship between variables. The significance levels of the tests was considered less than *p* < 0.05.

## Results

In total, out of 242 samples included in the study, 169 (69.8%) were nursing students, and the rest were midwifery students. The mean age of the participants in this study was 21.66 ± 2.25. A total of 180 (74.4%) of the participants were women, and the rest were men. A total of 237 (97.9%) of the participants were single.

The results of this study showed that the overall scores of clinical education status (103.16 ± 19.21) was at an average level. In the separate evaluation of the fields in the clinical education status questionnaire, the highest score belongs to the field of goals and education planning, with a score of 34.39 ± 6.66. It was obtained at an average level. The fields of the instructor's performance (mean: 32.35 ± 5.93), interacting with students (mean: 11.79 ± 3.13), the educational environment (mean: 12.81 ± 4.46) and monitoring and evaluation (mean: 11.80 ± 3.45) were placed at average levels. Additionally, in the separate examination of the fields in the questionnaire of factors affecting effective clinical education, the highest score was reported in the field of personal characteristics of the student, with a score of 33.97 ± 5.99. In addition, the mean scores of the fields of personal characteristics of the instructor clinical, the conditions of the clinical environment, educational planning, and clinical evaluation were obtained with means of 22.66 ± 4.28, 17.25 ± 5.11, 21.29 ± 5.46 and 20.80 ± 5.79, respectively.

In the separate evaluation of the fields in the clinical education status questionnaire in nursing students, the goals and educational program were on the average level (mean: 33.34 ± 6.61). In this group, in the separate evaluation of the fields in the questionnaire of factors affecting effective clinical education, the highest score was reported in the field of personal characteristics of the student (mean: 33.69 ± 6.49) (Table 1). In midwifery students, in the clinical education status questionnaire, the highest score was reported in the field of goals and educational planning (mean: 36.82 ± 6.18). Additionally, in the questionnaire of factors affecting effective clinical education in this group, the highest score was in the field of personal characteristics of the student (mean: 34.60 ± 4.61), which was on the average level (Table 2).

**Table 1:** Mean and standard deviation of nursing students scores

Questionnaire	Fields	Mean	Std. Deviation
Clinical Education Status	Goals and Educational Program	33.34	6.61
	Instructor's Performance	32.12	6.22
	Interacting with Students	11.50	3.06
	Educational Environment	12.09	4.32
	Monitoring and Evaluation	11.66	3.51
	Overall score	100.73	18.97
Factors Affecting Effective Clinical Education	Personal Characteristics of The Student	33.69	6.49
	Personal Characteristics of The Clinical Instructor	22.73	4.56
	Clinical Environment Conditions	17.14	5.44
	Educational Planning	21.16	5.83
	Clinical Evaluation	20.80	6.03
	Overall score	115.55	24.97

**Table 2:** Mean and standard deviation of midwifery students scores

Questionnaire	Fields	Mean	Std. Deviation
Clinical Education Status	Goals and Educational Program	36.82	6.18
	Instructor's Performance	32.89	5.21
	Interacting with Students	12.45	3.22
	Educational Environment	14.47	4.38
	Monitoring and Evaluation	12.13	3.30
	Overall score	108.78	18.69
Factors Affecting Effective Clinical Education	Personal Characteristics of The Student	34.60	4.61
	Personal Characteristics of The Clinical Instructor	22.52	3.57
	Clinical Environment Conditions	17.50	4.27
	Educational Planning	21.58	4.51
	Clinical Evaluation	20.80	5.25
	Overall score	117.02	19.68

Considering the size of the samples of each group, there was no need to check the hypothesis of normality and use non-parametric tests. Comparing the scores of the clinical education status between the two groups of nursing and midwifery students using the t test of two independent samples showed that these scores were significantly higher in midwifery students than in nursing students (*p* = 0.003). However, there was no significant difference in the score of factors affecting effective clinical education between the two groups (*p* = 0.623) (Table 3). For performing multivariate analysis, we first compute the Pearson’s correlation coefficient to check if these 2 variables are correlated. The Pearson’s correlation coefficient is 0.399 (*p* =  < 0.001) which show a significant and positive linear relationship between these 2 variables. So, more factors affecting effective clinical education, more the clinical education status. For modelling the clinical education status respect to factors affecting effective clinical education and other covariance, we used a multiple linear regression model (Table 4).

**Table 3:** T-test for statistical difference between nursing and midwifery students

Independent Samples Test										
		Levene's Test for Equality of Variances		t-test for Equality of Means						
		F	Sig	t	df	Sig. (2-tailed)	Mean Difference	Std. Error Difference	95% Confidence Interval of the Difference	
									Lower	Upper
Clinical Education Status *p* = 0.003 Reject H0	Equal variances assumed	.006	.938	-3.03	240	.003	-8.04	2.64	-13.25	-2.82
	Equal variances not assumed			-3.05	138.56	.003	-8.04	2.63	-13.24	-2.83
Factors Affecting Effective Clinical Education *p* = 0.623 Accept H0	Equal variances assumed	6.989	.009	-.44	240	.654	-1.47	3.29	-7.96	5.00
	Equal variances not assumed			-.49	171.39	.623	-1.47	2.99	-7.39	4.44

**Table 4:** Coefficients of variables predicting clinical education status

Coefficients^a^						
Model		Unstandardized Coefficients		Standardized Coefficients	t	Sig
		B	Std. Error	Beta		
1	(Constant)	68.02	6.83		9.94	.000
	Factors Affecting Effective Clinical Education	.32	.04	.40	7.12	.000
	Field of Study	6.44	2.36	.15	2.72	.007
	Academic Semester	-2.35	.57	-.23	-4.13	.000

^a^ Dependent Variable: Clinical Education Status

The results of the Kruskal‒Wallis test showed that there is a significant relationship between the scores of the general state of clinical education among different entries (*p* = 0.001). However, there is no significant difference between the scores of factors affecting effective clinical education among different entries (*p* = 0.624). This test also showed that there is a significant relationship between the general state of clinical education and interest in the field of study (*p* =  < 0.040). However, no significant relationship was found between the scores of factors affecting effective clinical education and interest in the field of study (*p* = 0.015).

The results of the present study showed that age has no significant relationship with the general state of clinical education (*p* = 0.585 *r* = -0.035) or predictive factors of effective clinical education (*p* = 0.424 r =  + 0.052). The Pearson correlation coefficient between the grade point average and the effective factors on effective clinical education was reported to be equal to + 0.149, and a significant relationship between these two variables was obtained (*p* = 0.020); however, the overall grade point average had no significant relationship with the state of clinical education (*p* = 0.740 *r* = -0.021).

## Discussion

The current study, which was conducted to investigate the clinical education status and determine the effective clinical education factors from the point of view of nursing and midwifery students, showed that clinical education is at an average level. This finding is in line with the results of some studies in Iran [12, 20, 21]. The study by Qin et al. in China showed that the understanding of specialist nurses with a master's degree about the quality of educational services based on the clinical environment is at an average level [22]. Although 41.5% of the study samples of Qin et al. had clinical work experience and a master's degree, the results of the above study and the present study are consistent. Contrary to the results of the present study, the samples of Ebadi et al.'s study have stated that clinical education is at a good level [16]. In Ebadi et al.'s study, which was conducted among public and private university students, private university students had a better view than public university students. It seems that the difference between facilities in private universities compared to public universities can be one of the reasons for the best condition of clinical education. Therefore, the views of students at private universities influenced the general results of the above study and caused the difference between the results of the above study and the present study. In another study in South Africa, Jaganath et al. showed that nursing students' satisfaction with the clinical learning environment is in a favorable state [23]. It seems that the facilitating factors of clinical education in this country have increased the level of satisfaction with the clinical learning environment by influencing the students' views in this field.

### Fields of clinical education status

Among the fields of clinical education status, the field of "educational objectives and planning" was placed on the average level despite obtaining the highest score. This finding is in line with the findings of Emami et al.'s study, which was conducted on private and public nursing students in Yazd city [24]. The findings of Zadi et al.'s study also report this result [25]. Contrary to the results of the present study, in the study of Seidi et al., this field was reported at a good and appropriate level [26]. Variable clinical environments with different educational facilities, as well as the formulation of realistic goals at the beginning of educational planning at this university, can be the reasons for the difference in results. Apart from this, in the study of Seidi et al., in addition to nursing and midwifery students, surgical technology students were also included in the study.

In the current study, the field of "Instructor's Performance" was also at an average level, which is in line with the results of some studies [24, 27, 28]. However, the study of Taylan et al. in Turkey showed that the instructor's performance is at a good level from the point of view of nursing students [29]. In this study, the high level of support among clinical instructors and effective communication with students with a positive effect on students' views has placed the status of this field at a high level. Contrary to the results of the present study, in the study of Kol et al., among first-year nursing students, the performance of the clinical instructor was reported to be good [30]. It seems that the access to clinical instructors, the enthusiasm of the instructors for training, and allocating enough time for the students in the lower semesters have caused this positive approach toward the instructors. In addition, it seems that students in the first year of study have different knowledge and attitudes compared to students in higher semesters, and this issue can explain the difference between the results of the above study and the present study.

In the current study, the fields of "interacting with students" and "monitoring and evaluation" were also at an average level, which is in line with the results of some studies [16, 26, 31]. Contrary to the results of the present study, Aghaei et al., in a study on nursing students, reported the status of these two fields as poor [27]. Perhaps one of the reasons for the difference in the results of the two studies is the lack of the same evaluation system in the research environments of the two studies. In addition, the results of the present study showed that the field of "Educational Environment" is at an average level, which is in line with the results of some studies [12, 16, 26]. Contrary to the results of the present study, Aghaei et al. reported this field at a poor level in their study [27]. Inappropriate behavior of medical personnel in the community studied by Aghaei et al., lack of amenities, and lack of attention to student needs are among the influential factors in this finding.

### Factors affecting effective clinical education

In the present study, the field of personal characteristics of the student obtained the highest score and seems to have the greatest impact on effective clinical education. This finding is in line with the findings of Tang et al. and Khemtizare et al. [12, 32]. Unlike the present study, in the study of Attia et al. [33] among nursing students in Iraq, this field has a lower average than other fields. Perhaps one of the reasons for the difference in the results of the two studies can be attributed to the cultural background and different personality characteristics of the students. Apart from this, in the present study, the field of personal characteristics of the clinical instructor was one of the other fields that obtained high scores, which is in line with the results of the study of Attia et al. [33]. Although this field is ranked second in the present study, contrary to the results of the present study, Zadi et al. reported in their study among students of surgical technology and anesthesia that the clinical instructor has the greatest influence on the clinical education of these students [25]. This difference in the results may be due to the difference in the nature of these two fields compared to the nursing and midwifery fields. Inocian et al. showed in a review study that the clinical instructor has the greatest influence on the attitude of nursing students toward the clinical environment among different fields [34]. The studies that were analysed in this review are all from studies outside of Iran, and it seems that educational programs and clinical environments different from Iran can be among the reasons for the difference. Mortazavi et al.'s study in Arak showed that the clinical instructor has less influence on the quality of clinical education of students compared to other cases [35]. Although the above study was conducted using a mixed method, it seems that the main reason for the difference in the results of the two studies is the participant samples. In Mortazavi et al.'s study, the samples were from all fields of medical sciences, while in the present study, only nursing and midwifery students were included. The different natures of different fields of medical sciences can cause different information to be collected from the students of each field. On the other hand, the supervision of clinical instructors and the hours of presence of instructors with students in different disciplines are not the same.

In the present study, the two fields of "educational planning" and "clinical evaluation" obtained lower scores than the two fields of "personal characteristics of the student" and "personal characteristics of the clinical instructor". This result was also reported in the studies of KhemtiZare et al. and Taheri et al. [12, 19]. Contrary to the results of the present study, in the study of Jalalvandi et al., the effect of these two fields was reported more than in other fields [36]. Jalalvandi et al.'s study was conducted on medical students. It seems that different educational planning and clinical evaluations in the field of medicine caused the difference between the results of the above study and the present study. In the current study, the conditions of the clinical environment have the least effect on effective clinical education. The results of this study are in line with the results of Emami et al.'s study conducted on nursing students in Yazd city [24]. Contrary to the results of the present study, Isaacs et al., in a study on factors affecting clinical learning, showed that the clinical environment has a great impact on learning [37]. This study was conducted on medical graduate students. As mentioned, the time that students in this field spend in the clinical environment is longer than the time that nursing and midwifery students spend at the bedside. In another study, Chen et al. showed that the clinical environment is one of the most influential factors in the clinical education of nursing students in Taiwan [5]. It seems that the difference in the nursing education system in Taiwan compared to Iran caused the difference in the results. The nursing students of this country, according to the existing education system, go through a 7-year course to obtain a bachelor's degree. Because of this, they will spend more time in this environment, and this issue can affect the attitude of these students.

Additionally, the results of this study showed that clinical education is in a better state from the point of view of midwifery students compared to nursing students. Hasanian et al.'s study also showed that there is a significant difference between the nursing and midwifery groups in terms of understanding the educational environment, although Hasanian et al. used a different tool [38]. However, to some extent, the results of the two studies are consistent. Finding the cause of the difference in nursing and midwifery students are very important. This difference can be attributed to society's greater attention to midwifery. In addition, this group of students can work independently in private clinics after graduation and hope for a better future. The field of nursing is not well-known in society, and the services provided by nurses in different environments are still not well-known in society and in health organizations. The perception of nursing is still mostly influenced by the same stereotypical image. Although the field of nursing has bachelor's, master's, and doctoral degrees and has various nursing fields, it is still not well-known [38]. In this study, an inverse and significant relationship was reported between academic semester and the clinical education status. Basiri Moghadam et al. also reported that lower semester students report a better clinical education situation than higher semester students [39], which is in line with the findings of our study. Mahyar et al. also reported this finding in their study [20]. Contrary to the results of the present study, Mahdavian et al. reported in their study on nursing and midwifery students that there is a direct relationship between an academic semester and clinical training [40]. It should be noted that the number of samples in this study was less than the present study. Considering the difference in educational structures and educational facilities in private universities compared to state universities and the difference in teaching style in different universities, this result can be justified. On the other hand, L'Ecuyer et al. and Asirifi et al. reported that nursing students in higher semesters have a better clinical education status [41, 42]. Perhaps one of the reasons for the difference between the above studies and the current study can be attributed to the difference in educational structures, the type of clinical education provided to students, and the availability of facilities in these countries. The results of this study showed that there is a direct and significant relationship between the clinical education status and the factors affecting effective clinical education. The results of this study are in line with the results of the study by Ebadi et al. and the study by Mahyar et al. [16, 20].

### Strengths

One of the strengths of this research are the use of students' views as the main beneficiaries of clinical education trends and policies.

### Limitations

One of the limitations of the study are the transferability of the results because the structure and trends of clinical training in different environments have differences.

## Conclusion

The findings of this study showed that the clinical education status of nursing and midwifery students at Qazvin University of Medical Sciences is at an average level. Although all fields of clinical education were at an average level, among them, the field of goals and educational planning had the highest scores compared to other fields. In addition, it was found that the personal characteristics of the student have the most effectiveness on clinical education. According to the results, managers and policymakers are expected to provide a suitable platform for improving clinical education status by considering the factors affecting effective clinical education. Related professionals are expected to take steps toward improving the status of clinical education by adopting appropriate teaching methods. It is suggested that in future studies, a comparative study of variables between faculty members and students should be performed. The results of the present study can be used in similar environments.

## Data Availability

Data are available by contacting the corresponding author.
